# Dynamic profiling of medulloblastoma surfaceome

**DOI:** 10.1186/s40478-023-01609-7

**Published:** 2023-07-10

**Authors:** David Bakhshinyan, Yujin Suk, Laura Kuhlmann, Ashley A. Adile, Vladimir Ignatchenko, Stefan Custers, William D. Gwynne, Andrew Macklin, Chitra Venugopal, Thomas Kislinger, Sheila K. Singh

**Affiliations:** 1 McMaster Centre for Discovery in Cancer Research, McMaster University, MDCL 5027, 1280 Main Street West, Hamilton, ON L8S 4K1 Canada; 2 Department of Biochemistry and Biomedical Sciences, Faculty of Health Sciences, McMaster University, Hamilton, ON Canada; 3 Michael G DeGroote School of Medicine, McMaster University, Hamilton, ON Canada; 4 Princess Margaret Cancer Center, UHN, Toronto, ON Canada; 5 Department of Medical Biophysics, University of Toronto, Toronto, ON Canada; 6 Department of Surgery, Faculty of Health Sciences, McMaster University, Hamilton, ON Canada

**Keywords:** Medulloblastoma, Proteomics, Integrins

## Abstract

Medulloblastoma (MB) is the most common type of malignant pediatric brain cancer. The current standard of care (SOC) involves maximal safe resection and chemoradiotherapy in individuals older than 3 years, often leading to devastating neurocognitive and developmental deficits. Out of the four distinct molecular subgroups, Group 3 and 4 have the poorest patient outcomes due to the aggressive nature of the tumor and propensity to metastasize and recur post therapy. The toxicity of the SOC and lack of response in specific subtypes to the SOC underscores the urgent need for developing and translating novel treatment options including immunotherapies. To identify differentially enriched surface proteins that could be evaluated for potential future immunotherapeutic interventions, we leveraged N-glycocapture surfaceome profiling on Group 3 MB cells from primary tumor, through therapy, to recurrence using our established therapy-adapted patient derived xenograft model. Integrin 𝛼5 (ITGA5) was one of the most differentially enriched targets found at recurrence when compared to engraftment and untreated timepoints. In addition to being enriched at recurrence, shRNA-mediated knockdown and small molecule inhibition of ITGA5 have resulted in marked decrease in proliferation and self-renewal in vitro and demonstrated a survival advantage in vivo. Together, our data highlights the value of dynamic profiling of cells as they evolve through therapy and the identification of ITGA5 as a promising therapeutic target for recurrent Group 3 MB.

**Supplementary Information:**

The online version contains supplementary material available at 10.1186/s40478-023-01609-7.

## Introduction

Brain tumors have become the leading cause of pediatric cancer-related mortality surpassing hematological malignancies [1], with medulloblastoma (MB) representing the most common malignant brain tumor in children. Despite therapeutic advances, roughly 30–40% of MB patients succumb to disease recurrence and survivors often face long-term neurological and neurocognitive deficits [2]. In the past decade, multi-omic profiling of large cohorts of MB samples led to stratification of this disease into four distinct molecular subgroups, WNT, SHH, Group 3, and Group 4 [3, 4]. Each molecular subgroup displays a distinct gene expression profile, clinical characteristics, and prognosis. Building on the initial transcriptomic and genomic characterization, recent proteomic and phosphoproteomic analyses further underscore the existing heterogeneity in molecular drivers in MB, even among tumors belonging to the same subgroup [5, 6]. Of the 4 molecular subgroups, Group 3 MB tumors are endowed with increased incidence of tumor recurrence, leptomeningeal dissemination, and the worst 5-year overall survival [7]. This may be in part due to their propensity to harbor focal *MYC* amplifications reportedly occurring in 10–17% of G3 MB patients [8]. Therapy failure and consequentially disease recurrence is the single most adverse event in MB pathogenesis, which remains nearly universally fatal despite the improvements in adjuvant interventions. There is an urgent need for the development of novel, targeted therapies tailored for MB recurrence to improve patient outcomes and negate the long-term neurotoxic sequalae caused by intensive craniospinal irradiation and systemic chemotherapies.

Advancements in immunotherapy-based treatments led to drastic improvements in survival rates for leukemic cancers from 57% in the 1960 and 1970 s to 92% in 2012 [9]. Earlier studies leveraging chimeric antigen receptor T cell (CAR T) technology, have shown a remarkable response in acute lymphoblastic leukemia and non-Hodgkin lymphomas reducing recurrence rates to up to 80% and provided a strong rationale for the exploring its therapeutic potential against pediatric brain tumors [10, 11]. While there exists a diverse array of immunotherapeutic modalities in addition to CAR T cell therapy; including oncolytic virotherapy, NK-based cellular therapies, and dendritic cell vaccines – they all are dependent on the identification of tumor-specific or tumor-associated antigens that can be targeted for immune cell mediated cell death [12, 13]. Notably, there is a significant lack of cell surface targets that are both unique to recurrent MB and amendable to immunotherapy. The paucity of matched primary-recurrent MB samples is an important contributing factor to this unmet need. Previous studies have shown that effective targets for tumor clearance with manageable toxicity in preclinical models exhibit [14, 15].

Cell surface proteins play vital roles in cell-cell/cell-environment communication, signal transduction, and transportation of solutes across the plasma membrane, processes which are known to be dysregulated in cancer [16]. Moreover, cell surface proteins are tractable targets for the development of novel therapies, as they are easily accessible, and possess extra-cellular epitopes that can be targeted by immunotherapies, even in the absence of a druggable domain [17]. Despite its importance, the cell surface proteome, or surfaceome, has been notoriously difficult to study. Cell surface proteins are poorly soluble, and their identification is often hindered by highly abundant and soluble intracellular proteins, which dominate most proteomic detection strategies [18, 19]. Surfaceome analysis therefore relies on techniques that enrich and solubilize cell surface proteins, while intracellular proteins are depleted [16, 20]. One of the techniques used to address this challenge is N-glycocapture, an enrichment technique that takes advantage of the fact that the majority (~ 90%) of cell surface proteins are estimated to be glycosylated, with ~ 90% of glycoproteins being N-glycosylated [21].

In this work, we leveraged our established therapy-adapted patient-derived xenograft model of Group 3 MB progression [22] combined with N-glycocapture profiling to investigate changes in expression of surface proteins through tumor engraftment, regression, and recurrence. Label-free quantification of MB samples isolated from brains of mice undergoing chemoradiotherapy led to the identification of 23 proteins that were highly expressed (log10 fold-change > 5) in relapse when compared to the samples isolated at engraftment and untreated controls. Notably, the expression of only one such protein, integrin $\alpha$5 (ITGA5), was entirely absent in control and engraftment tumor samples, increased through chemoradiotherapy, and peaked at relapse.

The integrin family of proteins comprises 24 transmembrane proteins that are extensively involved in the mediation of cellular communication. Through binding to the extracellular matrix integrin signalling contributes to cytoskeletal organization and intracellular signalling that regulates cell survival, proliferation, migration, and fate determination [23]. The integrin signalling cascade is initiated upon binding of a ligand to integrin heterodimers, composed of alpha and beta subunits and is propagated intracellularly through a series of kinases. There is mounting evidence highlighting a role for ITGA5 in tumor progression, tumor metastasis, and therapy resistance across several malignancies including glioblastoma, colorectal carcinoma, pancreatic cancer, cervical cancer, and non-small cell lung carcinoma [24–30]. In this study, we further demonstrate the functional dependency of Group 3 MB on ITGA5 through shRNA- mediated knockdown studies and small molecule inhibitors. The combined proteomic profiling and molecular characterization of ITGA5-regulated cellular functions in recurrent Group 3 MBs provides a foundation for the development and validation of immune-based treatment modalities against this newly identified MB cell surface target.

## Methods

The experiments were not randomized, and investigators were not blinded to allocation during experiments and outcome assessment.

***Human MB cell culturing.*** All cell lines used were cultured in NeuroCult Complete (NCC) media: NeuroCult™ NS-A Basal Medium supplemented with 50mL NeuroCult™ Supplement (StemCell™ Technology #05750), 20ng/mL EGF, 10ng/mL FGF, 0.1% heparin and 1% penicillin-streptomycin for a minimum of 48 h prior to experimentation. SU_MB002 (RRID: CVCL_VU79) is a therapy refractory cell line derived from a patient treated with cyclophosphamide and displaying expression markers of Group 3 MB [31]. HD-MB03 (RRID: CVCL_S506) was isolated from a patient with metastasized Group 3 MB prior to treatment [32] and propagated in NCC supplemented with 1% penicillin–streptomycin and 10% fetal bovine serum (FBS). D425-Med (D425, RRID: CVCL_1275) [33], a treatment-naïve MB cell line, was propagated in high glucose Dulbecco’s Modified Eagle Medium (DMEM, Life Technologies# 11965-118) supplemented with 1% penicillin–streptomycin, and 20% FBS. The matched recurrent versions of D425, HD-MB03 and SU_MB002 were generated through the established, previously published in vivo treatment protocol [22]. Human fetal neural stem cells (hNSCs) were isolated using a previously described protocol [34] and cultured in NCC media. HEK293T (RRID: CVCL_0063) were obtained from the American Type Culture Collection (ATCC) and grown in DMEM complete media supplemented with 1% non-essential amino acids and 10% FBS.

***Intracranial xenografting of MB and in vivo treatment protocol.*** All in vivo studies were performed according to McMaster University Animal Research Ethics Board (AREB) approved protocols. Intracranial injections and treatments were performed as previously described [35] Cell numbers sufficient to generate a measurable tumor burden were previously determined and are as follows: D425–1 × 10^4^, HD-MB03–1 × 10^6^ and SU_MB002–5 × 10^5^. The mice designated to receive treatment were subjected to 2 Gy of craniospinal irradiation using GammaCell 3000 irradiator 14 days post-engraftment. A week post radiation treatment, mice were treated with a single dose cisplatin (2.5 mg/kg), vincristine (0.4 mg/kg) and cyclophosphamide (75 mg/kg) were as previously described [22]. Mouse brains were collected at recurrence and isolated using a previously described protocol [34].

### Protein digestion and glycopeptide enrichment for mass spectrometric analysis

The glycocapture protocol was performed similarly as described by Cogger et al. [36]. Briefly, cells were lysed in PBS:TFE (50:50) using pulse sonication and subsequent incubation at 60 °C for 2 h, with vortexing every 30 min. Protein concentration was determined using the BCA assay (Pierce) according to the manufacturer’s instructions. Yeast invertase (SUC2) was added as an internal control at a ratio of 1pmol SUC2 per 1 mg total protein. Cysteines were reduced with DTT (5mM final concentration) at 60 °C for 30 min and alkylated with iodoacetamide (25mM final concentration) at room temperature for 30 min. Samples were diluted 5 times with 100mM ammonium bicarbonate (pH 8.0). Trypsin was added at a 1:500 ratio and protein digestion was performed overnight at 37 °C. The digestion was quenched using 0.5% formic acid (F.A.).

Tryptic peptides were desalted on C18 Macrospin columns (Nest Group), lyophilized and resuspended in coupling buffer (0.1 M CH_3_CO_2_Na, 0.15 M NaCl, pH 5.5). Glycan chains were oxidized using 10mM NaIO_4_ for 30 min in the dark and peptides were once more desalted using the C18 macrospin columns. The lyophilized oxidized peptides were resolubilized in coupling buffer and captured on hydrazide magnetic beads (Chemicel, SiMAG Hydrazide) for 12 h at room temperature. The coupling reaction was catalyzed by adding aniline (50mM) and was allowed to continue for an additional 3 h at room temperature.

The hydrazide beads containing the covalently coupled oxidized glycopeptides were thoroughly washed (2x coupling buffer; 5 × 1.5 M NaCl; 5x HPLC H_2_O; 5x CH_3_OH; 5 × 80% CH_3_CN; 3x H_2_O; 3 × 100mM NH_4_HCO_3_, pH 8.0) to remove unspecific binders. N-glycopeptides were eluted off the hydrazide beads using 5U PNGase F in 100mM NH_4_HCO_3_, at 37ºC overnight.

Eluted glycopeptides were recovered, and the hydrazide beads were additionally washed 2x with 80% CH_3_CN solution. Glycopeptides were desalted using C18 stage tips, eluted using 80% CH_3_CN, 0.1% F.A. and lyophilized. The purified glycopeptides were dissolved in 21µL 3% CH_3_CN, 0.1% F.A. Peptide concentration was determined using a NanoDrop 2000 (Thermo Fisher Scientific) spectrophotometer.

#### Mass spectrometric analysis and protein identification

Glycopeptides (1.5 µg) were loaded on a 50 cm ES803 column (Thermo Fisher Scientific). Peptides were separated using a 2-hour gradient, at 250nL/min flow, using the EasyLC1000 nano-liquid-chromatography system (Thermo Fisher Scientific). The chromatography system was coupled to an Orbitrap Fusion Mass Spectrometer (Thermo Fisher Scientific) and MS/MS data were acquired in a data dependent mode. The Orbitrap mass detector was used for the MS/MS acquisition, and the maximum injection time was set to 100 milliseconds for enhanced sensitivity.

The acquired raw data were analyzed by the Max Quant software (version 1.6.1.0), using the complete human proteome (version 2016.07.13 containing 42,041 sequences) downloaded from UniProt. Searches were performed with a maximum of two missed cleavages. Carbamido-methylation of cysteines was specified as fixed modification, while oxidation of methionine and deamidation of asparagine to aspartic acid (0.98Da mass shift) were specified as variable modifications. False discovery of peptides was controlled using a target-decoy approach based on reversed sequences, and the false discovery rate was defined as 1% at site, peptide, and protein levels.

#### Data analysis

Bioinformatic analysis was performed on the MaxQuant output file: Asn-_AspSites.txt using R. Only asparagine deamidation events identified with a localization probability of minimum 0.8 which were also part of the N-glycosylation N-[!P]-S/T sequon (N = asparagine; [!P] = any amino acid either than proline; S/T = serine or threonine at the + 2 site) were carried over for further analysis. Peptides that were identified in only one out of the three biological replicates were excluded. Peptide intensities were log-transformed and subsequently normalized against the NPVLAANSTQFRDPK SUC2 peptide, which was reproducibly detected in all samples. Protein intensities were calculated by averaging the respective peptide intensities.

The quantified data set was aligned with the cell surface glycoproteomes previously published in the Cell Surface Protein Atlas (CSPA) [37] and the surfaceome predictor SURFY [38] to identify cell surface glycoproteins with high confidence.

### RT-qPCR analysis

Total RNA was extracted from 2.5 × 10^5^ cells with Total RNA Purification Kit (Norgen, Cat#37500), with modified elution volume of 27µL. RNA concentration was determined using NanoDrop 2000 Spectrophotometer and 1000µg of RNA was used to synthesize complementary DNA (cDNA) using iScript cDNA SuperMix (Bio-Rad, Cat#1708841) and a C1000 Thermo Cycler (Bio-Rad) according to the manufacturer’s guidelines. RT-qPCR analysis was performed with PerfeCTa SybrGreen (Quanta Biosciences, Cat#95054-100) and CFX96 instrument (Bio-Rad). Quantification of gene expression was performed by using CFX manager 3.0 software, with GAPDH as housekeeping gene. The following qPCR primers were used to measure mRNA levels of ITGA5 (FWD 5’-ACCTCTGATGCCTGAGTCCT-3’, REV 5’-AGAAGTACCCAGACCCCTCC-3’ and GAPDH (FWD 5’-TGAACCACCAACTGCTTAGC − 3’, REV 5’-GGCATGGACTGTGGTCATGAG-3’).

### Lentivirus generation

Initial transfection using second generation lentiviral packaging vectors psPAX2 (RRID: Addgene_12260) and pMD2.G (RRID: Addgene_12259) were performed using protocol for Lipofectamine 3000 (Invitrogen, Cat#L3000015) in T75 flasks. The lentivirus containing media was collected after 48 h post transfection and ultracentrifuged at 25,000 rpm for 2 h at 4 °C following resuspension in 1mL DMEM.

### Lentiviral titer calculation

HEK293T cells were seeded into 12-well plates at a density of 100,000 cells/well in 200µL of complete media. 100µL of concentrated virus was diluted in complete media to a final volume of 200µL (1:2), of which 20 µL was serially diluted 10-fold to a final concentration of 1 × 10^− 11^ in 200µL (1:10) and. Next, 50µL of each dilution was added to each well (1:5) in duplicate. An untransduced puromycin kill control was included. After 48 h, media was replaced with 2mL complete media containing 1 µg/mL of puromycin. After 4 days of puromycin selection and outgrowth, the number of colonies was enumerated where appropriate. For optimal accuracy, we calculated titers from the most diluted wells that still contain individual colonies. Viral titers were determined using the formula: *CFU = mean number of colonies × DF*, where CFU is colony forming units and DF represents the dilution factor. MB cells were transduced at MOI = 1 based on determined MOI values (shGFP = 2.0 × 10^7^ CFU/mL; shITGA5-1 = 5.0 × 10^7^ CFU/mL; shITGA5-4 = 2.5 × 10^8^ CFU/mL).

### Lentiviral transductions

2.5 × 10^5^ cells/well MB cells were seeded in a 6-well plate with 450µL of NCC media and 50µL lentiviral supernatant. An additional 500 µL of NCC was added 8 hours post transduction and 1mL of NCC added 24 hours post transduction. After 48hours, the cells underwent complete media change and were expanded with puromycin containing media for additional 48hours. Lentiviral pLKO.1 vectors expressing shRNA targeting human ITGA5 ((shITGA5-1 5’-CCATGATGAGTTTGGCCGATT-3’; shITGA5-2 5’-CCACTGTGGATCATCATCCTA-3’; shITGA5-3 5’-CCTCAGGAACGAGTCAGAATT-3’; shITGA5-4 5’-CTCCTATATGTGACCAGAGTT-3’) and vector expressing control GFP (shGFP 5’-ACAACAGCCACAACGTCTATA-3’) were contributed by Dr. Jason Moffat. ITGA5 knockdowns were validated by RT-qPCR and flow cytometry.

### Flow cytometric analysis

To evaluate surface levels of ITGA5 using MoFlo XDP (Beckman Coulter), single cell suspension in PBS + 2mM EDTA were stained with anti-CD49e antibody (1:1000, Biolegend, Cat#328,011) for 15 min at room temperature followed by staining with viability dye, 7-AAD (1:10, Beckman Coulter) for 5 min.

### Cell proliferation

MB cells were sorted into a 96-well plate at a density of 1000 cells/well in 200µL NCC in quadruplicates. After a 72-hour incubation period, 20µL PrestoBlue Cell Viability Reagent (Life Technologies) was added to the cells. Fluorescence was measured after 4 h at excitation and emission wavelengths of 540-570 nm respectively with the FLUOstar Omega Microplate reader (BMG Labtech) and analyzed using the Omega software.

### Self-renewal and in vitro limiting dilution assay

MB cells were sorted at a density of 200 cells/well in 200µL NCC with 6 technical replicates per samples. After 72 h of incubation, each well was evaluated for the number of spheres containing 7 or more cells. Self-renewal was defined as number of spheres per 500 cells. The in vitro limiting dilution was evaluated by seeding MB cells in a 96-well plate at densities ranging from 1000 cells/well to 1 cell/well in 200µL of NCC. Four technical replicates per dilution were plated. After a 72-hour incubation period the percentage of wells without spheres were scored and plotted against the number of cells/well. The dilution with 1 self-renewing cell was determined based on dilution corresponding to the fraction of negative wells equal to 0.37 [39].

### Dioscin in vitro dose-response curves

1000 cells/well were seeded in a 96-well plate at a volume of 200 µL with dioscin (Cayman, Cat#19057-60-4) and diluted 1.25-fold in quadruplicates to concentrations from 200nM to 26.84nM. Wells treated with DMSO served as controls. Proliferation was measured using PrestoBlue Cell Viability Reagent (Life Technologies) after 72-hour of incubation and IC_50_ was determined by plotting log_10_ transformed concentration of Dioscin versus the percentage of viable cells. IC_80_ values were calculated using IC(F) = [(100-F)/F]^1/HS^ × IC_50_ where F represents percent reduction of proliferation and HS is the calculated hill slope.

### Statistical analyses

At least three technical or experimental replicates from each experiment were compiled. Data represent mean ± standard deviation with n values listed in figure legends. GraphPad PrismTM was used to plot all bar graphs and statistical analyses including student’s t-test or 2-way ANOVA, p < 0.05 was considered significant. All Kaplan-Meier survival plots were plotted with GraphPad PrismTM and long-rank (Mantel-Cox) test was performed for comparison of median survival, p < 0.05 was considered significant.

## Results

### N-glycocapture based surfaceome profiling of MB cells through therapy

**Fig. 1:**
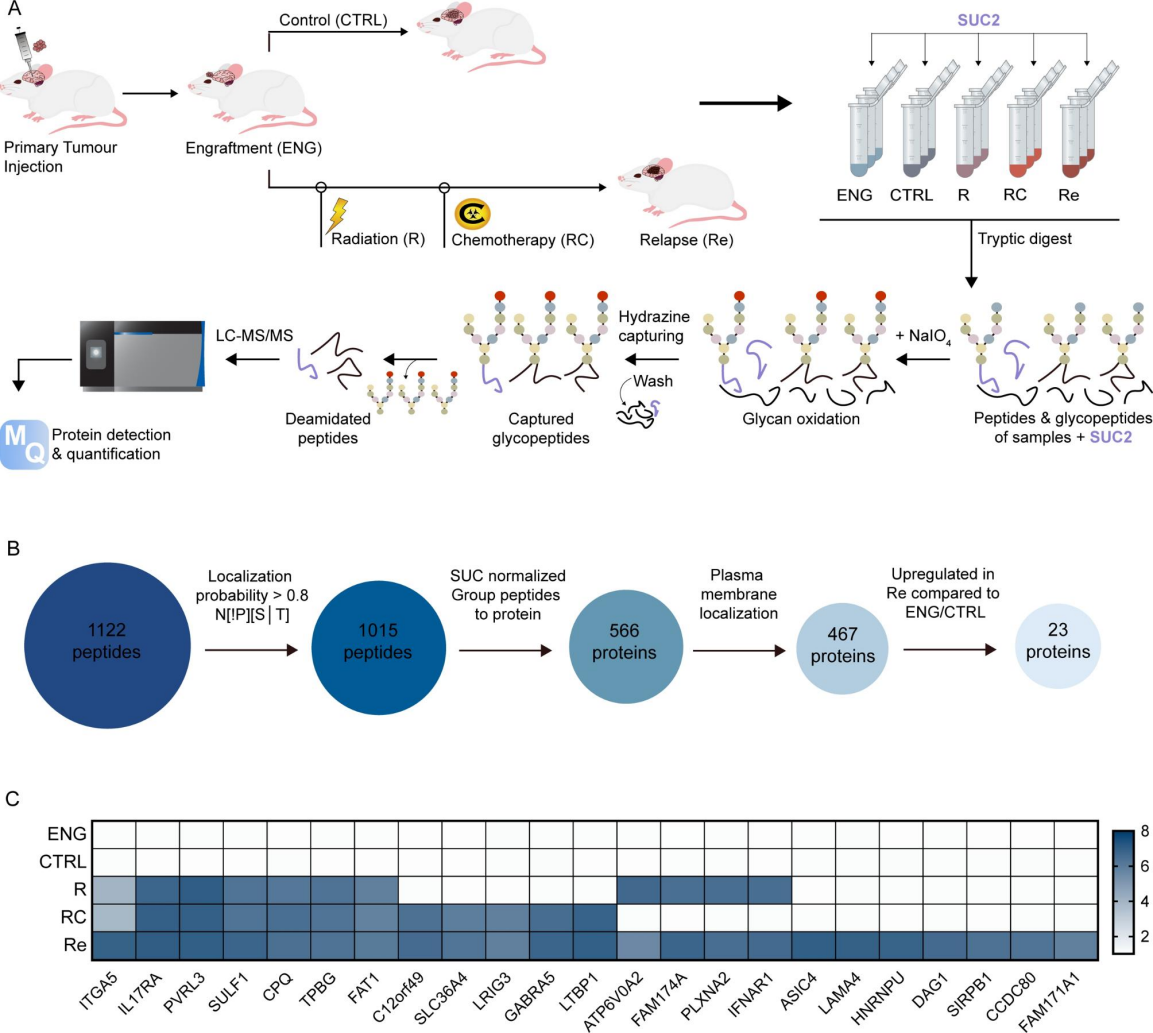
Identification of ITGA5 as a highly expressed cell surface glycoprotein in Group 3 MB relapse. **(A)** Schematic representation of the glycocapture enrichment protocol of patient-derived human Group 3 MB samples. Established PDX mouse-adapted therapy model enabled isolation of brain samples at engraftment (ENG), control (CTRL), post-radiation (R), post-radiation and chemotherapy (RC), and relapse (Re). **(B)** Sequential steps to identify cell surface proteins enriched at relapse. **(C)** Top 14 cell surface glycoproteins highly expressed in relapse, but not expressed in engraftment and control samples; common logarithmic (Log10) scale used

We used our established therapy-adapted PDX model of MB recurrence [22] to profile MB tumors through radiation, chemoradiation, and at recurrence. Briefly, human derived Group 3 MB cells were intracranially xenografted into immunocompromised mice and treated with the established chermoradiotherapy regimen. Mice bearing Group 3 MB tumors that received the combined chemoradiotherapy treatment demonstrated an initial tumor burden regression and an extended survival. However, all treated animals inevitably showed signs of recurrence with brain tumor burden and extent of leptomeningeal dissemination exceeding those found in the untreated animals. Functional profiling of cells isolated through therapy revealed an increasingly aggressive phenotype with cells isolated at recurrence demonstrating increased proliferation and self-renewal capacities. Relevant to this work, collection of MB cells from mouse brains at engraftment (ENG), post-radiation (R), post-radiation and chemotherapy (RC), and relapse (Re) as well as from untreated controls (CTRL) allowed for comparative analysis of surfaceome of MB cells through therapy. The glycocapture protocol (Fig. 1A) lead to the detection and quantification of 1015 N-glycopeptides, mapping to 566 proteins (Fig. 1B, Supplementary Tables 1, Additional File 1). SUC2, a yeast glycoprotein with no significant sequence homology to human glycoproteins was used as an internal control, to account for potential variations in the efficiency of the enrichment protocol. The intensity profiles of deamidated sites (N-sites) and the SUC2 internal standard were similar across all samples, underlining the high reproducibility of the employed workflow (Supplementary Fig. 1A, B). Furthermore, unsupervised clustering of label-free quantified data revealed close clustering of biological triplicates and distinct proteomic profiles of cells isolated at each stage through therapy. (Supplementary Fig. 1C). We focused our attention on 467 glycoproteins with a confirmed cell surface localization, as determined by data mining [37, 38]. The surface glycoproteome list was further restricted to proteins with an intensity > 5 log10 in the three relapse samples, which were also absent in the control (CTRL) and engraftment (ENGR) samples. Of the top 23 proteins enriched at relapse (Fig. 1C, Supplementary Fig. 1D, Supplementary Fig. 2), ITGA5 was the only candidate whose expression rose steadily from engraftment to relapse suggesting treatment-driven selection and subsequent expansion and was thus chosen for further investigation. Additionally, *in silico* mining of the available microarray data from HD-MB03 MB cells undergoing mouse-adapted in vivo chemoradiotherapy [22] revealed an increased mRNA expression of ITGA5 at relapse when compared to tumors samples isolated at engraftment prior to treatment and at endpoint of untreated controls (Supplementary Fig. 1E). To assess whether the increase in ITGA5 has further biological implications, we probed surface expression of integrin ß1 (ITGB1), the main binding partner of ITGA5 required for downstream signalling and found it to be 10-fold higher than the levels detected at engraftment and 100-fold higher compared to the untreated controls (Supplementary Fig. 1F). As a tumor primarily arising in the developing cerebellum, MB cells often depend on similar molecular pathways as healthy human neural stem cells (hNSCs). The major drawback of current gold-standard therapies for MB is their indiscriminatory nature in targeting both malignant MB cells and healthy NSCs, leading to long-term neurocognitive deficits in survivors. Comparison of ITGA5 signal intensities in NSCs and MBs showed a significant difference (Supplementary Fig. 1G), which indicates a potential therapeutic window for ITGA5-directed therapies.

### ITGA5 expression marks MB cell population with an increased self-renewal ability

To further investigate the clinical relevance of upregulated ITGA5 expression in MB, we probed the publicly available gene expression profiles of a large cohort of MB patients with the accompanying clinical parameters including overall survival, subgroup affiliation, and patient age [3]. Elevated mRNA expression of ITGA5 was found to be predictive of lower overall survival in patients under the age of 18 diagnosed with Group 3 or Group 4 MBs (Supplementary Fig. 3A). Additionally, ITGA5 levels appeared to be higher in Group 3 MB, a *MYC*-driven subtype associated with the increased frequency of tumor recurrence and leptomeningeal dissemination, when compared to other subtypes (Supplementary Fig. 3B). Furthermore, the early onset of MB also correlated with increased expression of ITGA5 (Supplementary Fig. 3C).

**Fig. 2:**
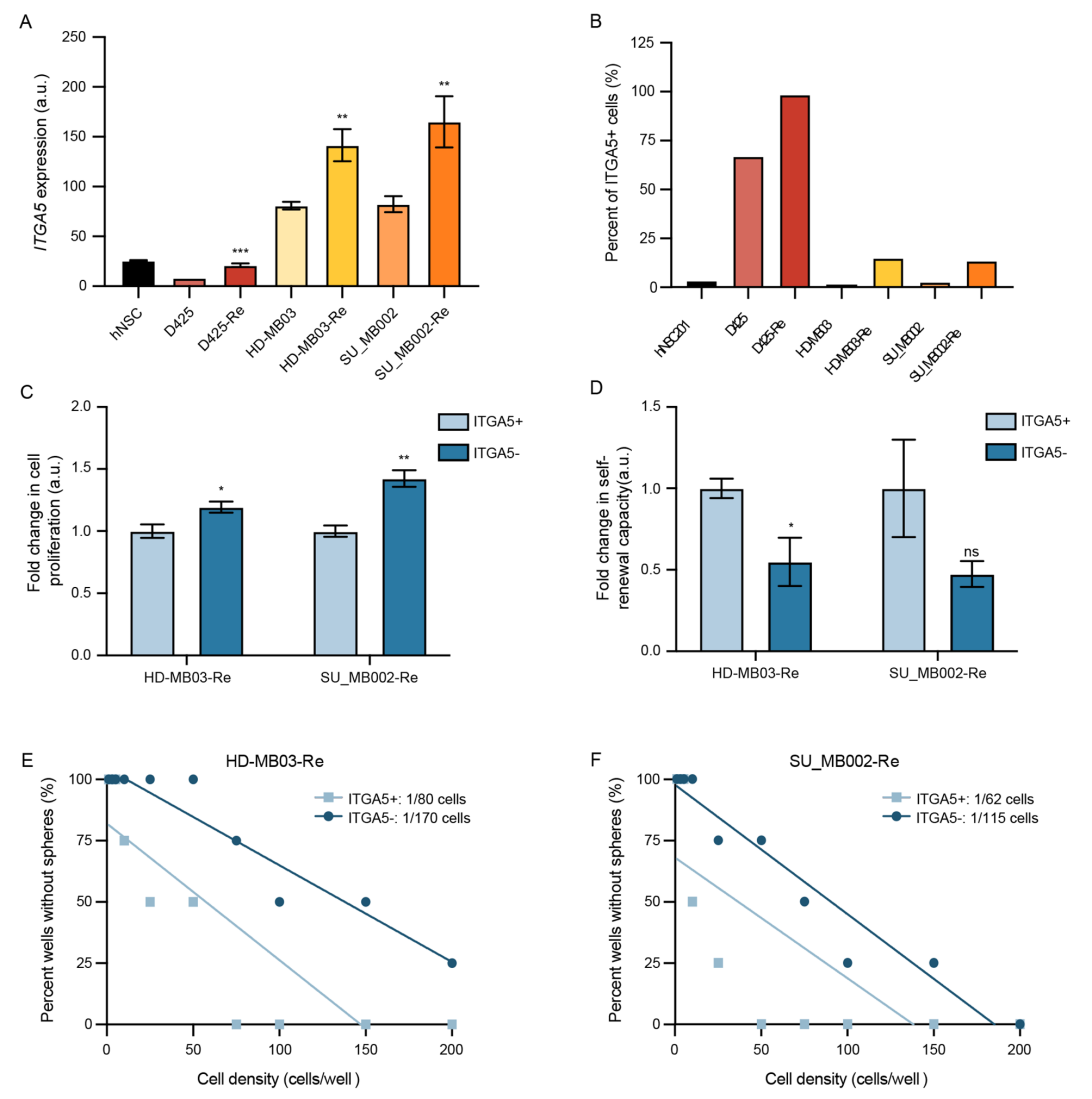
Increased ITGA5 expression correlates with aggressive phenotype of Group 3 MB. **(A)***ITGA5* mRNA levels and **(B)** flow cytometric evaluation of cell surface levels of ITGA5 in human neural stem cells (hNSCs), as well as primary and matched-recurrent MB lines. Changes in **(C)** proliferation and **(D)** self-renewal capacity of recurrent Group 3 MB cells with (ITGA5+) and without (ITGA5-) ITGA5 presenting on the cell surface. Fraction of self-renewing cells in **(E)** HD-MB03-Re and **(F)** SU_MB002-Re post flow based on ITGA5 presentation on the cell surface. Bars represent mean of at least three technical replicates, with the exception of Fig. 2B with only one value. Bars represent mean of at least three technical replicates. *p ≤ 0.05, **p ≤ 0.001, ***p ≤ 0.0001, ****p ≤ 0.00001; unpaired t-test or one-way ANOVA with Sidak’s method for multiple comparisons

To validate our initial observation of increased ITGA5 in Group 3 MB recurrence, we probed 2 additional matched primary and generated recurrent tumors, D425 vs. D425-Re and SU_MB002 vs. SU_MB002-Re, for *ITGA5* mRNA levels through RT-qPCR and surface expression of ITGA5 through flow cytometric profiling. In all 3 pairs, we observed a marked increase in *ITGA5* mRNA (Fig. 2A) and cell surface protein expression levels (Fig. 2B). Next, we set out to explore the functional differences in ITGA5-expressing (ITGA5+) compared to non-expressing (ITGA5-) fractions of recurrent Group 3 MB cells through established stem cell assays. While cells in the ITGA5 + fraction appeared to have lower proliferation (Fig. 2C), they were observed to have increased self-renewal capacity as assessed through sphere formation assays (Fig. 2D) and limiting dilution assays (Fig. 2E, F).

### shRNA and small molecule-based targeting of ITGA5 reduces the aggressive phenotype of recurrent G3 MB

**Fig. 3:**
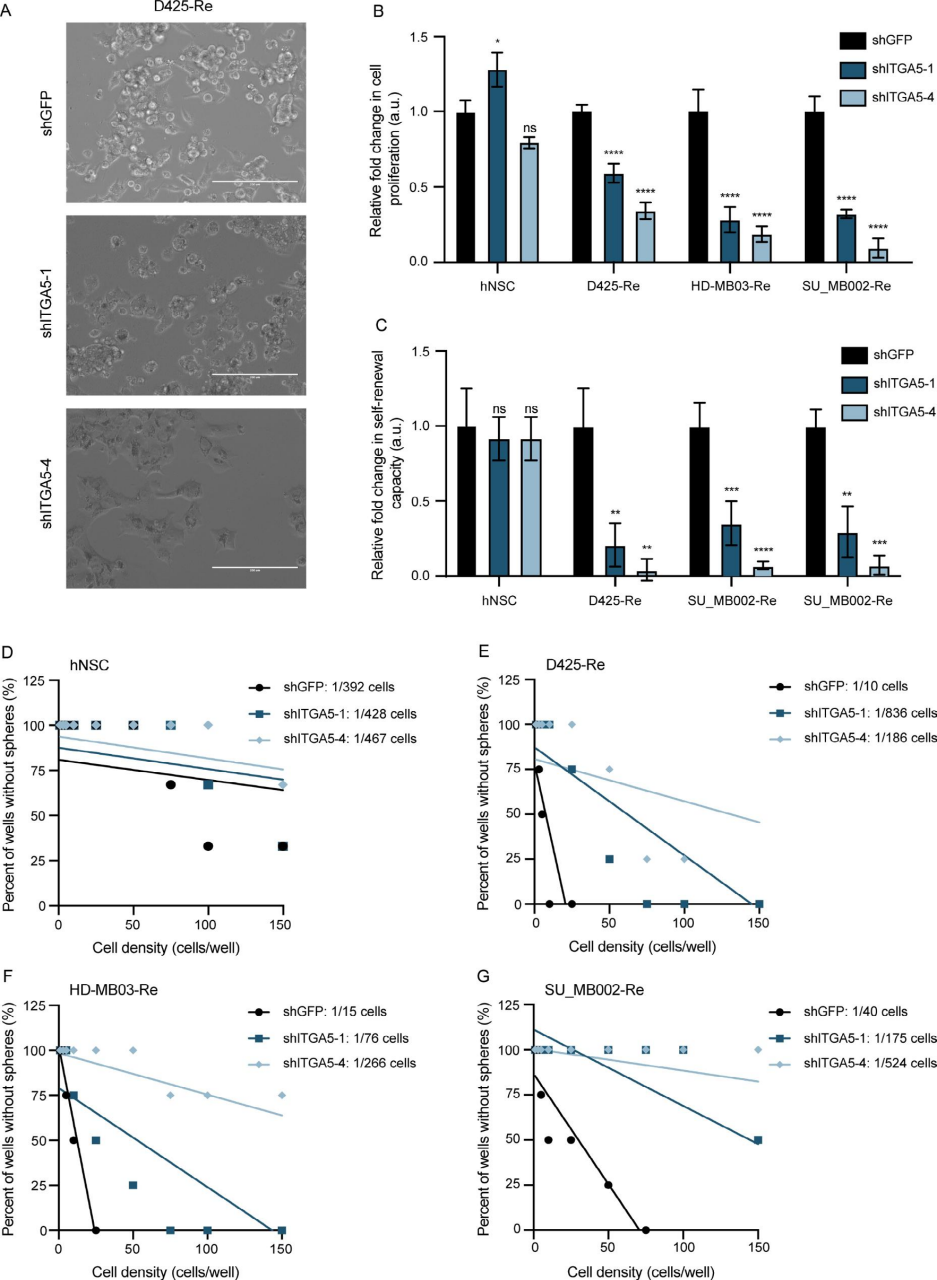
In vitro effects of ITGA5 KD in MB cell lines. **(A)** Microscopic images demonstrating phenotypic changes post lentivector mediated KD of ITGA5 in representative recurrent Group 3 MB. Changes in **(B)** proliferation, **(C)** self-renewal, and **(D-G)** frequency of self-renewing cells of ITGA5 KD, compared to control lentivector in hNSC and recurrent Group 3 MB cell cultures. Bars represent mean of at least three technical replicates. Control samples (shGFP) normalized to 1.0. Bars represent mean of at least three technical replicates. *p ≤ 0.05, **p ≤ 0.001, ***p ≤ 0.0001, ****p ≤ 0.00001; unpaired t-test or one-way ANOVA with Sidak’s method for multiple comparisons

**Fig. 4:**
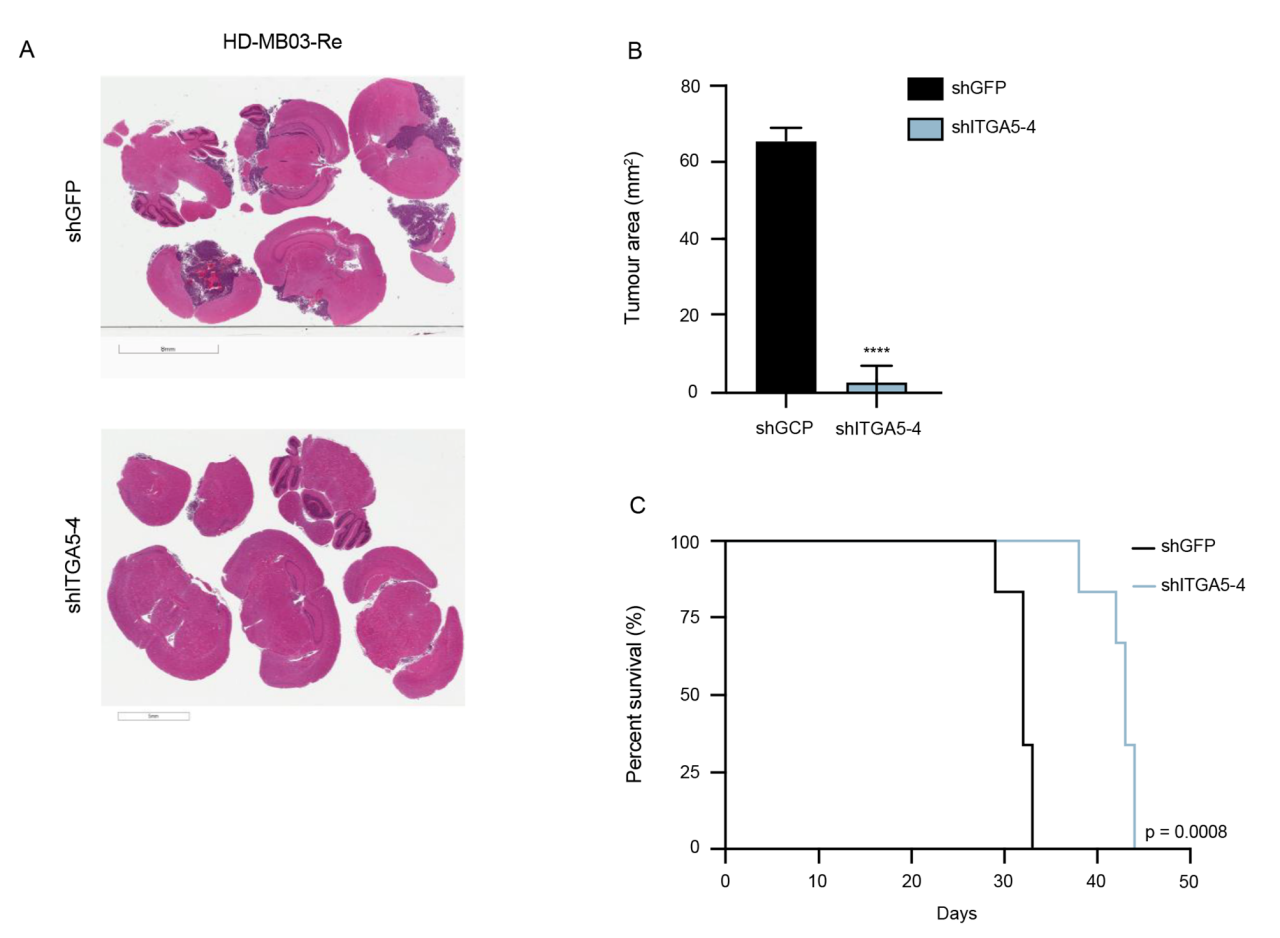
In vivo effects of ITGA5 KD in HD-MB03-Re. **(A)** Representative H&E-stained sections of brains isolated from mice xenografted with HD-MB03 lentivirally transduced with shITGA5-4 construct vs. shGFP (control). Samples were compared at endpoint of control animal to allow for time-matched comparison. **(B)** Quantification of tumor burden (mm^2^) of time-matched specimens **(C)** *Kaplan-Meier* survival of mice engrafted of control vs. ITGA5 KD in HD-MB03-Re.

**Fig. 5:**
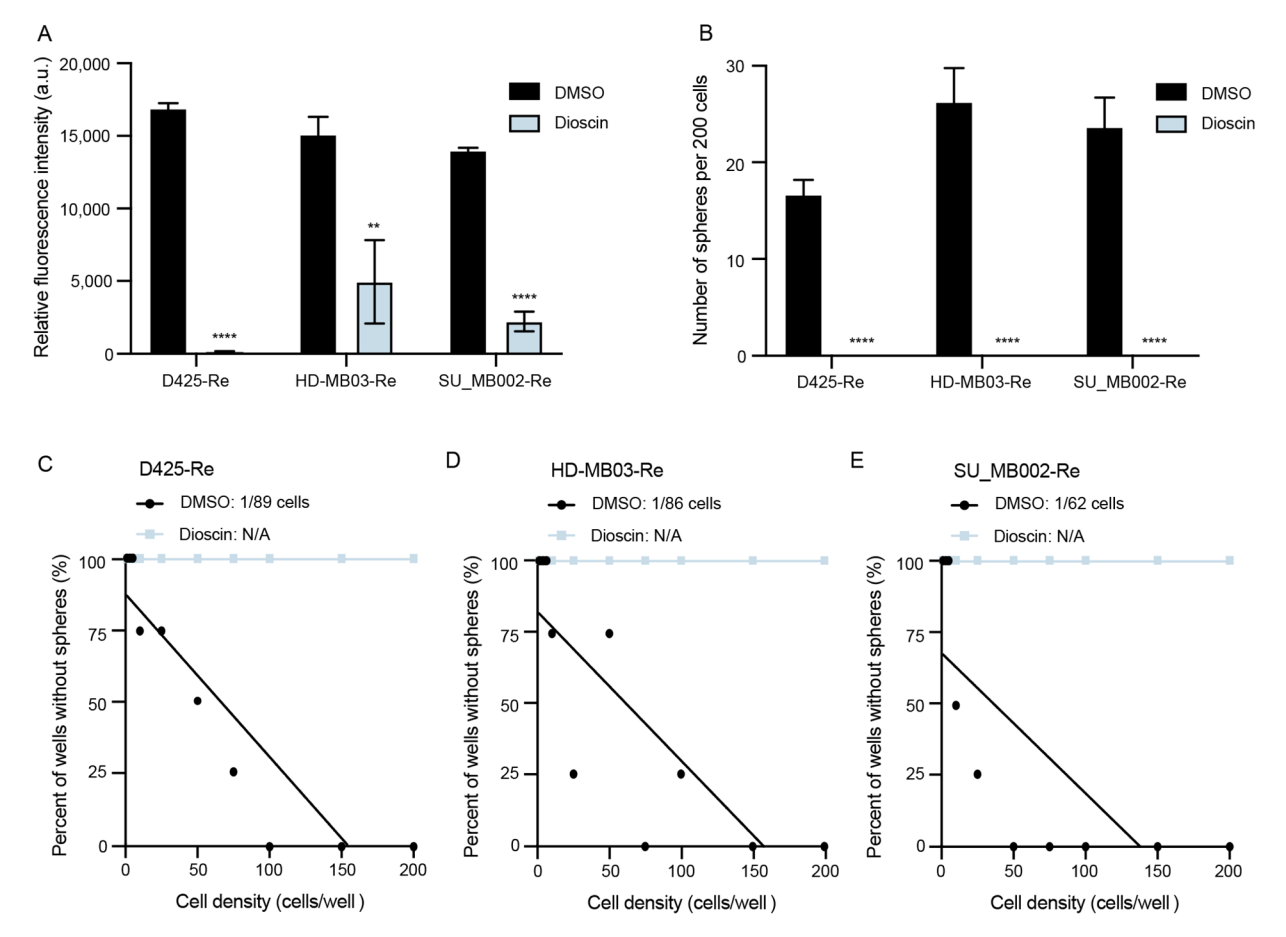
Potent ITGA5 inhibitor, dioscin, demonstrates efficacy against characteristic cancer stem cell properties. Inhibitory effect on **(A)** proliferation, **(B)** self-renewing potential, and **(C-E)** frequency of self-renewing cells in recurrent Group 3 MB cells. Bars represent mean of at least three technical replicates. Bars represent mean of at least three technical replicates. *p ≤ 0.05, **p ≤ 0.001, ***p ≤ 0.0001, ****p ≤ 0.00001; unpaired t-test or one-way ANOVA with Sidak’s method for multiple comparisons

To further probe the functional role of ITGA5 in recurrent Group 3 MB, we undertook functional studies using the 2 most efficient shRNA vectors targeting ITGA5 in 3 recurrent MB cell lines. The initial screen of 4 shRNA constructs in HEK293T cells (shITGA5-1, 2, 3, 4; Supplementary Fig. 4A) and subsequent viral transductions used for titer calculation showed no phenotypic changes in cell morphology, in any of the constructs used (Supplementary Fig. 4B). In contrast, lentiviral transduction of recurrent MB cells with shITGA5-1 and shITGA5-4 constructs exhibited extensive changes in cellular morphology (Fig. 3A), reduced proliferation (Fig. 3B), almost complete abrogation of self-renewal potential (Fig. 3C), and reduced fraction of self-renewing cells (Fig. 3F-G). Notably, we identified that *ITGA5* knockdown had no significant effects on human normal neural stem cells through in vitro validation of their self-renewal and proliferation capacity following shITGA5 transduction. (Fig. 3A, B, D). To validate the significant in vitro functional effects of *ITGA5* knockdown on brain tumor initiating capacity we xenografted HD-MB03-Re cells transduced with control and ITGA5-4 short hairpins and measured tumor burden (Fig. 4A, B) and overall survival (Fig. 4C). *ITGA5* KD significantly delayed engraftment and extended the overall survival by greater than 30% compared to control. The extent of *ITGA5* KD was validated by RT-qPCR (Supplementary Fig. 4C) and flow cytometry (Supplementary Fig. 4D). The promising antitumor effects of *ITGA5* KD observed through characteristic stem cell assays led us to further explore the therapeutic potential of ITGA5 through targeting it with small molecule-based inhibitor, dioscin [40]. Initial dose response curves were used to determine the half-maximal inhibitory concentrations (IC_50_) for each of the 3 recurrent Group 3 MB samples along with hNSCs. Surprisingly, IC_50_ values in all 4 samples were comparable, possibly due to additional molecular targets affected by dioscin (Supplementary Fig. 5A, B). Functionally, treatment of dioscin in the recurrent Group 3 MB cells led to a significant reduction of proliferation (Fig. 5A), and diminished self-renewal potential (Fig. 5B, C).

Given the functional importance of the members of the integrin family, including ITGA5, it is imperative to understand the expression levels across healthy tissues in order to gain insight into developing the novel therapeutics. Probing *ITGA5* mRNA and protein expression levels in publicly available data sets, revealed overall low to undetectable ITGA5 levels in healthy brain tissues [41, 42] (Supplementary Fig. 6), further corroborating results from the functional studies evaluating effect of ITGA5 KD in hNSCs.

## Discussion

Over the last 30 years, MB therapies have remained largely unchanged and although five-year survival rates have reached 70–80%, there remain patients who present with tumor relapse and/or metastatic dissemination thus, being limited to palliation. Therefore, there is an unmet need for novel target discovery pipelines in pediatric brain tumors that can focus on clinically relevant brain tumor initiating markers on cancer cells capable of evading therapy and driving tumor recurrence. The target identification pipeline and subsequent validation described in this work is specific to finding antigens on the cell surface that are recurrent-specific as they increase in abundance following selective pressures of chemoradiotherapy.

Protein glycosylation is the most common post-translational modification of extracellular proteins and contributes to a multitude of physiological processes. Up to 90% of surface proteins are predicted to be glycosylated, allowing for development of assays, including cell-surface capture (CSC) and N-glycocapture analysis, that use carbohydrate chains to isolate and subsequently identify plasma membrane proteins [16]. Two major classes of glycoproteins exist that are distinguished by the linkage between amino acid and sugar moieties. N-glycoproteins represent 90% of predicted glycoproteins, with the remaining 10% made up of exclusively O-glycosylated proteins [21]. In the recent years, N-glycocapture technique has been increasingly utilized to identify potential therapeutic vulnerabilities including work focusing on breast cancer [43] and lymphoma cell lines [44], and glioblastoma [45]. One of the key limitations of N-glycocapture is the loss of subcellular localization resolution, rendering the need to confirm plasma membrane localization using additional methodology [16]. The unbiased screening of the Group 3 MB surfaceome allowed us to explore longitudinal changes in abundance of N-glycosylated proteins on the cell surface of Group 3 MB undergoing chemoradiotherapy in our established therapy-adapted model [22]. Of the identified proteins, ITGA5 was found to be one of the top differentially glycosylated proteins at the surface that uniquely increased in response to each additional therapeutic intervention. The apparent functional significance of ITGA5-modulated signalling in a tumor cell population selected through chemotherapy and radiation was validated in vivo and in vitro with significant reduction of self-renewal, a key stem cell property, as well as marked survival benefits associated with knockdown of ITGA5. Although functional assays of shRNA-mediated ITGA5 knockdown identified a reduction in both proliferation as well as self-renewal, cells that were sorted based on FACS-identified ITGA5 cell surface presence were observed to have increased proliferation with diminished self-renewal when compared to negative ITGA5 cells. This observation can likely be attributed to the dynamic changes in ITGA5 expression on the cell surface and thus signalling in the absence of permanent inhibition. The increased self-renewal capacity of ITGA5 positive cells despite a lower proliferation rate may be indicative of its functional significance in subpopulation of Group 3 MB cells capable of therapy evasion and driving tumor recurrence.

The role of integrins in cancer has been extensively documented in existing literature. As a member of the integrin family, ITGA5 has been shown to play a regulatory role in tumor cell proliferation, migration, invasion, ECM remodeling, angiogenesis, therapy evasion and modulating immune infiltration across multiple cancer types and models including other types of brain tumors [26, 27, 30, 46, 47]. We attribute recurrent-specific markers such as ITGA5 as likely to target tumor subpopulations that are responsible for tumor metastatic programs, angiogenic features, and those that are prone to exhibit therapy evasion with increased self-renewal capacities as identified in our in vitro self-renewal assays [46]. However, further investigation is needed to elucidate the exact mechanisms behind these programs in Group 3 MB. Some of the results generated in other cancers have demonstrated ITGA5 to exert its effect by modulating PI3K/AKT and MAPK/ERK [48].

To date, several targeting modalities against ITGA5 have been developed, including small molecule (dioscin) [40], a humanized monoclonal antibody (volociximab) [49] and a peptidomimetic (AV3) [24]. While validated as an ITGA5 inhibitor by a docking assay, dioscin, may contribute to ITGA5-independent modulation of PI3K, AKT and mTOR signalling pathways, explaining the narrow therapeutic window in Group 3 MB when compared to hNSCs. In turn, this further emphasizes the need to identification of more precise therapeutic interventions.

The therapeutic potential of immunotherapy and CAR T cell therapy for pediatric brain cancers is an emerging field with promising preclinical and clinical findings indicating their potential to treat aggressive, treatment refractory tumors while mitigating toxic effects [11, 14]. Group 3 MB patients often present with leptomeningeal metastases at diagnosis that have limited therapeutic modalities and is currently considered to be incurable with current SOC. Unlike challenges with brain-blood-barrier permeability and often narrow therapeutic window affecting small molecule-based interventions, the recently published reports of efficacious locoregional delivery of immunotherapeutics against pediatric brain tumors [50], make an ITGA5-specific immunotherapy-based treatment a promising intervention against recurrent Group 3 MB. While the apparent functional significance of ITGA5 in recurrent Group 3 MB and limited expression on the surface of healthy brain tissue make ITGA5 a promising targeting, the redundancy of large integrin family may pose a therapeutic challenge if ITGA5 is targeted alone. To date, several factors have been identified in stifling the success of immune-cell based therapies including cancer specific antigen selection, antigen downregulation through the course of therapy or even antigen loss [51]. To overcome these challenges, several new approaches have been developed allowing for co-targeting of multiple antigens using “OR”, “AND” and “NOT” Boolean logic gates [52].

Historically, the approach to personalized medicine has been through application of ever improving gene sequencing technologies. While next-generation sequencing (NGS) of tumor genome has led to identification of actionable genes in one third of the patients, only a small fraction of those had documented clinical benefit [53, 54], highlighting the limitation of only relying on genomic and transcriptomic profiling. Therefore, there has been a growing shift towards a multi “-omic” approach where the tumor samples are assessed across multiple levels of biology to generate an integrated overview of underlying pathology. Proteomics has become the tool for assessment of protein expression, their localization and post-translational modifications to allow for discovery of novel molecular vulnerabilities to be exploited in hopes of generating a meaningful clinical improvement. The combination of cell surface proteomic profiling and in vivo therapy-adapted models present a discovery pipeline that is suitable for identifying clinically relevant markers for solid cancers and specifically pediatric brain tumors that are known to be cold tumors with a paucity of neoantigens amenable to therapeutic targeting. Subsequent in vitro validation in the proposed cells of origin such as human neural stem cells with Group 3 MB can lead to development of immunotherapeutic modalities that may spare the vulnerable developing brain from the neurotoxic sequelae stemming from the cytotoxic standard of care therapies [55].

### Electronic supplementary material

Below is the link to the electronic supplementary material.

Supplementary Material 1: Fig. 1, related to Fig. 1 Glycocapture box blot **(A)** prior to and **(B)** after SUC2-normalization (yellow dot; NPVLAANSTQFRDPK peptide). **(C)** Hierarchical clustering heatmap based on Euclidean distance of differentially expressed proteins demonstrating close clustering of technical triplicates. **(D) ITGA5** peptide tag intensity, **(E)***ITGA5* mRNA expression and **(F)** ITGB1 protein expression in Group 3 MB cells isolated at pre-determined timepoints through therapy. **(G)** Comparison of ITGA5 intensity to SUC2 demonstrating selective enrichment for ITGA5 in Group 3 MB through therapy. Bars represent mean of at least three technical replicates. *p ≤ 0.05, **p ≤ 0.001, ***p ≤ 0.0001, ****p ≤ 0.00001; unpaired t-test or one-way ANOVA with Sidak’s method for multiple comparisons. Fig. 2, related to Fig. 1. **(A)** A Venn diagram demonstrating the number of unique and overlapping surface proteins enriched at each stage of therapy when compared to their expression at engraftment and control timepoints. **(B)** A list of proteins used to generate the Venn diagram. R = post-radiation; RC = post-chemoradiotherapy; Re = relapse. Fig. 2, related to Fig. 2. In silico evaluation of ITGA5 in publicly available MB repository. **(A)** Kaplan-Meir curve demonstrating worse overall survival in patients (n = 288) with relative mRNA expression of *ITGA5* over 4.6 (RMA- Normalized). **(B)** Transcriptional expression of *ITGA5* of 632 patients across 12 MB subtypes described in Cavalli et al. and **(C)** based on age group affiliation. **p ≤ 0.001; one-way ANOVA with Dunnett’s method for multiple comparisons. Fig. 3, related to Fig. 3. Characterization and validation of ITGA5 KD. **(A)** mRNA expression of *ITGA5* in HEK293FT cells post ITGA5 KD. **(B)** Microscopic images of HEK293FT cells post ITGA5 KD. Changes in **(C)***ITGA5* mRNA expression in reccurent Group 3 MB cells post ITGA5 KD. **(D)** Flow cytometric evaluation of changes in ITGA5 surface expression in HD-MB03-Re cells post ITGA5 KD. Bars represent mean of at least three technical replicates. *p ≤ 0.05, **p ≤ 0.001, ***p ≤ 0.0001, ****p ≤ 0.00001; unpaired t-test or one-way ANOVA with Sidak’s method for multiple comparisons. Fig. 4, related to Fig. 4. Selectivity characterization of dioscin in recurrent Group 3 MB cells and hNSCs. **(A)** Dose response curves and **(B)** corresponding IC_50_ concentrations and hill slopes of dioscin in hNSCs and three recurrent Group 3 MB lines. Points represent mean of three technical replicates, normalized to DMSO. Error bars represent standard error of the mean. IC_50_ and Hill slope values standardized to two decimal places. Fig. 5, related to Fig. 4. Expression of ITGA5 in healthy human tissue samples **(A)** ITGA5 protein levels as detected by whole cell proteomics in health tissues, reported by Wang et al. Protein intensity is reported as intensity-based absolute quantification (iBAQ) values, normalized using median centering across tissues. **(B)** ITGA5 protein expression as detected by antibody staining using the Human Proteome Atlas public repository. Staining strength corresponds to expression levels, including high (3), medium (2), low (1), and not detected (0). **(C)** mRNA expression of *ITGA5* in various tissues according to Genotype-Tissue Expression (GTEx) dataset (https://www.proteinatlas.org/); expressed in transcripts per million (TPM).

## Data Availability

The datasets supporting the conclusions of this article are included within the article (and its additional file).
