# Correction to: Dynamic profiling of medulloblastoma surfaceome

**DOI:** 10.1186/s40478-023-01666-y

**Published:** 2023-10-23

**Authors:** David Bakhshinyan, Yujin Suk, Laura Kuhlmann, Ashley A. Adile, Vladimir Ignatchenko, Stefan Custers, William D. Gwynne, Andrew Macklin, Chitra Venugopal, Thomas Kislinger, Sheila K. Singh

**Affiliations:** 1 Centre for Discovery in Cancer Research, McMaster University, 1280 Main Street West, MDCL, Hamilton, ON 5027, L8S 4K1 Canada; 2 2Department of Biochemistry and Biomedical Sciences, Faculty of Health Sciences, McMaster University, Hamilton, ON Canada; 3 3Michael G DeGroote School of Medicine, McMaster University, Hamilton, ON Canada; 4 Margaret Cancer Center, UHN, Toronto, ON Canada; 5 5Department of Medical Biophysics, University of Toronto, Toronto, ON Canada; 6 6Department of Surgery, Faculty of Health Sciences, McMaster University, Hamilton, ON Canada

**Correction to**: ***Acta Neuropathologica Communications *(2023) 11:111**

10.1186/s40478-023-01609-7

Following publication of the original article [1], the authors identified an error in the author names of the third and the eighth author.

The incorrect author names are: Laura Kuhlman and Andrew Mackling.

The correct author names are: Laura Kuhlmann and Andrew Macklin.

The author group has been updated above and the original article [1] has been corrected.
